# Gas-phase reaction mechanism in chemical dry etching using NF_3_ and remotely discharged NH_3_/N_2_ mixture†

**DOI:** 10.1039/d0ra05726f

**Published:** 2020-08-20

**Authors:** Akira Matsugi, Shiro Kubota, Yuichi Funato, Yutaka Miura, Kazuhiko Tonari

**Affiliations:** National Institute of Advanced Industrial Science and Technology (AIST) 16-1 Onogawa Tsukuba Ibaraki 305-8569 Japan a.matsugi@aist.go.jp; Institute of Advanced Technology, ULVAC, Inc. 2500 Hagisono Chigasaki Kanagawa 253-8543 Japan; Institute of Advanced Technology, ULVAC, Inc. 1220-1 Suyama Susono Shizuoka 410-1231 Japan

## Abstract

Modeling of dry etching processes requires a detailed understanding of the relevant reaction mechanisms. This study aims to elucidate the gas-phase mechanism of reactions in the chemical dry etching process of SiO_2_ layers which is initiated by mixing NF_3_ gas with the discharged flow of an NH_3_/N_2_ mixture in an etching chamber. A kinetic model describing the gas-phase reactions has been constructed based on the predictions of reaction channels and rate constants by quantum chemical and statistical reaction-rate calculations. The primary reaction pathway includes the reaction of NF_3_ with H atoms, NF_3_ + H → NF_2_ + HF, and subsequent reactions involving NF_2_ and other radicals. The reaction pathways were analyzed by kinetic simulation, and a simplified kinetic model composed of 12 reactions was developed. The surface process was also investigated based on preliminary quantum chemical calculations for ammonium fluoride clusters, which are considered to contribute to etching. The results indicate the presence of negatively charged fluorine atoms in the clusters, which are suggested to serve as etchants to remove SiO_2_ from the surface.

## Abstract 2

Reactions of NF_3_ and a discharged NH_3_/N_2_ mixture generate etchant species for destroying SiO_2_ layers.

## Graphical abstract

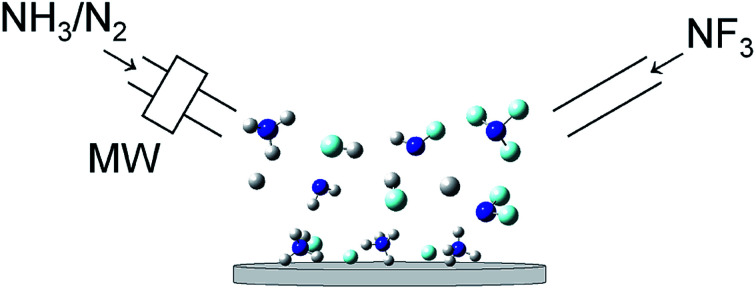

## 1. Introduction

Dry etching of materials is one of the key components in the fabrication of sophisticated semiconductor devices. Chemical dry etching utilizing remote plasma^1–4^ is a reliable and promising technique for damage-free etching and has mainly been applied to the removal of native oxides (SiO_2_) from silicon surfaces. The continuous scaling down of circuit dimensions requires narrower and deeper contact patterns, which are difficult to create with the conventional wet process. In this regard, dry processes are suitable for high-aspect ratio etching and have many other advantages, such as uniformity and selectivity, over wet cleaning processes.

**Fig. 1:**
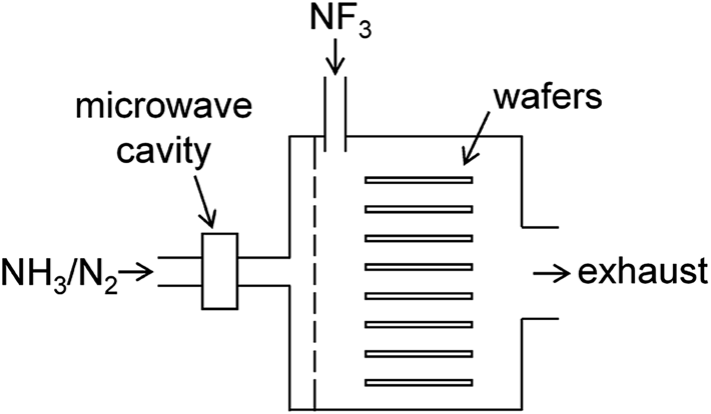
Schematic diagram of the chemical dry etching apparatus.

The present study focuses on chemical dry etching using NF_3_ gas and remotely discharged mixture of NH_3_ and N_2_.^2–4^Fig. 1 shows a schematic diagram of an equipment used in this process. The wafers are placed in a batch-type reactor and exposed to etchant gases produced by the gas-phase reactions. A mixture of NH_3_ and N_2_ gases is introduced into the chamber *via* an inlet port after passing through the microwave cavity where radical species are generated by the microwave discharge at 2.45 GHz. Pure NF_3_ gas supplied *via* the other inlet port reacts with the radicals in the chamber to generate etchant species. After etching, the wafer surface is covered by some deposited compounds, which are considered to be (NH_4_)_2_SiF_6_ and possibly contain ammonium fluorides.^1,5^ These residues are removed by heating the substrates in a post processing procedure.

The performance of the etching process, including the etching rate and selectivity, is highly dependent on the process parameters such as gas flow rates, pressure, and temperature; therefore, elucidation of the etching mechanism is warranted to understand the phenomena and have better control of the process conditions. However, the reaction mechanism of the etching process has yet to be understood. Based on their observations in etching experiments using NH_3_/NF_3_ downflow plasma, Nishino *et al.*^1^ proposed that negatively charged F atoms in ammonium fluorides preferentially attack Si atoms in SiO_2_ layers, leading to the substitution of the Si–O bond with the Si–F bond by a mechanism similar to the wet etching of SiO_2_ using a hydrogen fluoride solution. This mechanism presumes the formation of ammonium fluoride on the SiO_2_ surface, which would be generated from NH_3_ and HF adsorbed on the surface. Because HF is a product that should be formed in the gas-phase reactions of NF_3_ and hydrogen-containing species, obtaining a good understanding of the gas-phase reaction mechanism is an important first step to reveal the whole mechanism. Along with this line, Hayashi *et al.*^5^ reported the results of quantum chemical calculations of the NF_3_ + H and subsequent reactions, and suggested a potential reaction scheme for the formation of HF; however, the feasibility of the proposed reaction pathways has not yet been kinetically investigated.

In the present study, a kinetic model representing the gas-phase reactions in the chemical dry etching is constructed. The model is based on the reported reaction mechanism of the combustion of nitrogen-containing species,^6–8^ and the reaction pathways and rate constants for the primary reactions are updated or newly evaluated by quantum chemical calculations and statistical reaction rate theories. Because reactions occurring in the microwave discharge are difficult to quantitatively predict, the types of radicals produced from discharged NH_3_/N_2_ mixtures are deduced based on available literature data, as described later. Then, the reactions of the radicals with NF_3_ and potential subsequent reactions are investigated and implemented in the model. An example of a kinetic simulation using the constructed model is presented for a representative reaction condition. The results of the preliminary calculations of (NH_3_)_*n*_(HF)_*m*_ clusters and their implications for the surface process are also presented.

## 2. Computational methods and conditions

Quantum chemical calculations were performed using the Gaussian 09 (ref. 9) and Molpro 2018.2 (ref. 10) programs. Geometries of stationary points (reactants, products, intermediates, and transition states) were optimized using density functional theory with the ωB97X-D hybrid functional^11^ and the split-valence 6-311++G(d,p) basis set. Numerical integration of the exchange-correlation potential was performed with an ultrafine grid as implemented in Gaussian 09, having 99 radial shells and 590 angular points per shell. Harmonic vibrational frequencies were also calculated at the same level of theory. The zero-point energy (ZPE) and vibrational frequencies were scaled by 0.975 and 0.950, respectively,^12^ to approximately account for anharmonic effects. Single-point energies at the optimized structures were refined by an explicitly correlated coupled cluster method, CCSD(T)-F12b,^13–15^ with correlation-consistent polarized valence triple-ζ basis sets optimized for explicitly correlated methods, cc-pVTZ-F12.^16^ The ZPE-corrected ground-state energies are reported. For open-shell species, the spin unrestricted and restricted methods were used in the ωB97X-D and CCSD(T)-F12b calculations, respectively.

The rate constants were calculated by transition state theory based on the calculated energies and rovibrational properties using rigid-rotor and harmonic-oscillator approximations. The tunneling corrections were applied using one-dimensional approximation by assuming an asymmetric Eckart potential determined from the imaginary frequencies of the transition states.^17^ Some of the studied reactions involved chemically-activated intermediates, and the rate constants for these reactions were calculated by solving master equation^18,19^ using the SSUMES program.^20^

The rate constant calculations were performed for reactions involving H, NH_2_, NH, and N radicals, which are assumed to be produced from the microwave discharge of NH_3_/N_2_ mixture as follows. The etching rate is significantly reduced if pure NH_3_ gas, instead of the NH_3_/N_2_ mixture, is used for the radical source; this indicates that activated nitrogen is primarily responsible for the generation of radicals. There have been a number of studies on “active nitrogen”^21–24^ and its reactivity toward NH_3_ is considered to be dominated by the reaction of ^3^N_2_ + NH_3_,^23,24^ where ^3^N_2_ represents the A^3^Σ_u_^+^ state of N_2_. This reaction has been studied by several researchers,^25–28^ and found to proceed dominantly with the following two channels:

R1a ^3^N_2_ + NH_3_ → N_2_ + NH_2_ + H

R1b ^3^N_2_ + NH_3_ → N_2_ + NH + H_2_

with a total rate constant of ∼10^−10^ cm^3^ per molecule per s and a branching fraction of ∼0.9 for the former channel. Under typical operation conditions, the partial pressure of NH_3_ in the discharged flow is ∼100 Pa, and, therefore, ^3^N_2_ formed in the discharge rapidly reacts with NH_3_ on a microsecond timescale, which is much shorter than the radiative lifetime of ^3^N_2_.^29^ Atomic nitrogen, N(^4^S), can also be formed in the discharge, but is not reactive with NH_3_ and can survive for a long time before entering the chamber. Therefore, the radicals produced in these reactions, H, NH_2_, NH, and N, are considered to contribute to the gas-phase reactions in the chamber.

Based on the rate constant calculations, a kinetic model was constructed and used to perform a kinetic simulation to investigate the gas-phase reaction mechanism in the chamber. For simplicity, the simulation was performed for a homogeneous (zero-dimensional) and constant-pressure reactor using the Cantera program.^30^ The simulation condition was selected to roughly correspond to typical operation conditions of the etching process. The total pressure, *p*, was set to ≈200 Pa, consisting of 50 Pa of NF_3_, 100 Pa of N_2_, 50 Pa of NH_3_, and small amounts of the radicals. The gas temperature, *T*, in the chamber is nonuniform and considered to be higher near the inlet port because of the microwave discharge. However, because of a lack of detailed information on the temperature distribution, it was assumed to be constant at 350 K in the present simulation. The reaction duration was set to be close to the gas residence time in the chamber, ∼1 s.

The initial concentrations of the radicals are difficult to evaluate and are rather crudely estimated here as follows. The preliminary mass spectrometric analysis of gases in the etching chamber showed that the gas composition was dominated by the three reactants (NF_3_, N_2_, and NH_3_) and that signals from potential reaction products were difficult to quantify but were roughly two orders of magnitude smaller than those of the reactants. This indicates that only a few percent of the NH_3_/N_2_ mixture was converted to the radicals introduced into the chamber. Based on this assumption and the reported branching fractions of (R1a) and (R1b), the initial partial pressures of the H, NH_2_, and NH radicals in the chamber were set to be 1, 1, and 0.1 Pa, respectively. The formation of N atoms in the discharge is considered to be relatively minor compared to ^3^N_2_ because the dissociation asymptote of N(^4^S) + N(^4^S) lies ≈340 kJ mol^−1^ (3.54 eV) higher than the A^3^Σ_u_^+^ state of N_2_. If the electron-impact cross sections have similar values for the two excitation channels, the branching ratio for the N atoms can be estimated to be ∼exp(−3.54/*T*_e_), where *T*_e_ is the electron temperature. Here, a branching ratio of ∼0.1 was assumed and the initial partial pressure of N atoms was assumed to be 0.1 Pa.

For the calculations of (NH_3_)_*n*_(HF)_*m*_ clusters, the structures and frequencies were calculated using the same method as above, ωB97X-D/6-311++G(d,p), but the coupled cluster calculations with the triple-ζ basis sets were not affordable for large clusters; therefore, the energies of the clusters were calculated using the CBS-QB3//ωB97X-D method.^12,31^ The thermodynamic properties of the clusters were calculated using rigid-rotor and harmonic-oscillator approximations.

## 3. Results and discussion

### 3.1 Reaction pathways and rate constants

The reaction of NF_3_ with H atoms proceeds with the following two channels:

R2a NF_3_ + H → NF_2_ + HF

R2b NF_3_ + H → NHF_2_ + F

**Fig. 2:**
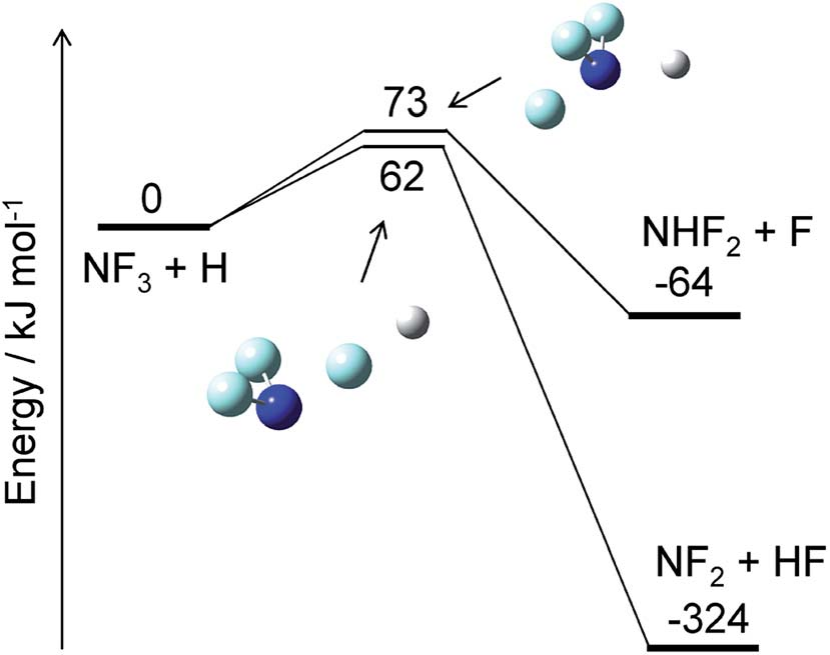
Energy diagram for the NF_3_ + H reaction and the structures of the transition states. The ZPE-corrected ground-state energies relative to the reactants are shown.

**Fig. 3:**
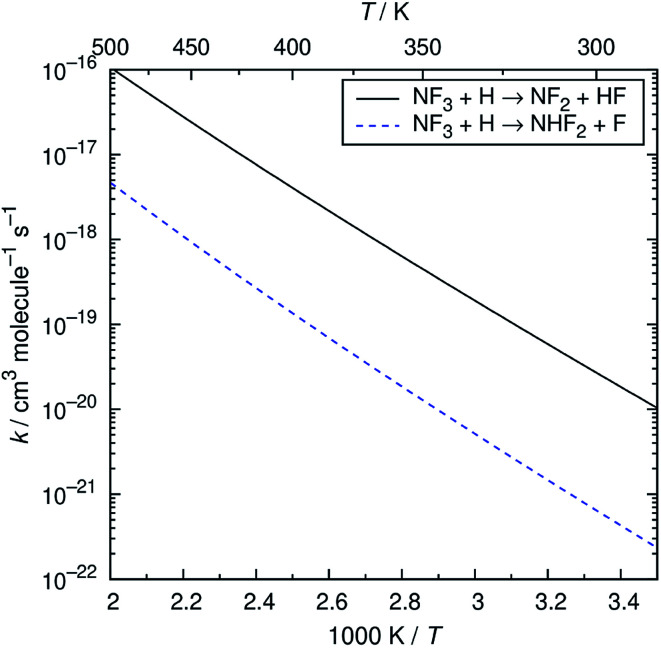
Arrhenius plot of the calculated rate constants for the NF_3_ + H reaction.

The energy diagram and the transition state structures are shown in Fig. 2. The fluorine abstraction channel (R2a) has a barrier height of 62 kJ mol^−1^, which is 11 kJ mol^−1^ lower than that of the F/H substitution channel (R2b). The rate constants, *k*, calculated for these two channels are shown in Fig. 3. As expected from the difference in the barrier heights, the abstraction channel has significantly larger rate constants than those of the substitution channel. The rate constants for (R2a) are 2.4 × 10^−20^ and 3.7 × 10^−19^ cm^3^ per molecule per s at temperatures of 300 and 350 K, respectively, whereas those for (R2b) are a factor of ≈30 smaller at these temperatures.

The reactions of NF_3_ with the other three radicals (NH_2_, NH, and N) also proceed with the fluorine abstraction and substitution mechanisms. The calculated barrier heights, reaction energies, and rate constants at 350 K are summarized in Table 1. The barrier heights for these radicals are all higher than 100 kJ mol^−1^, which results in rate constants that are many orders of magnitude smaller than those for H atoms. The rate constants on the order of 10^−29^ cm^3^ per molecule per s or less hardly compete with the reaction of NF_3_ with H atoms under typical conditions of the etching chamber; therefore, the reactions of NF_3_ with NH_2_, NH, and N are not implemented in the kinetic model presented later.

**Table 1:** Calculated barrier heights (Δ*E*_TS_), reaction energies (Δ*E*), and rate constants at 350 K for the NF_3_ + X (X = H, NH_2_, NH, and N), NHF_2_ + H, and NH_2_F + H reactions

Reactants	Products	Δ*E*_TS_/kJ mol^−1^	Δ*E*/kJ mol^−1^	*k* (350 K)/cm^3^ per molecule per s
NF_3_ + H	NF_2_ + HF	62	−324	3.7 × 10^−19^
NF_3_ + H	NHF_2_ + F	73	−64	9.5 × 10^−21^
NF_3_ + NH_2_	NF_2_ + NH_2_F	119	−43	3.2 × 10^−29^
NF_3_ + NH_2_	NF_2_NH_2_ + F	105	36	4.5 × 10^−29^
NF_3_ + NH	NF_2_ + NHF	130	−53	4.3 × 10^−31^
NF_3_ + NH	NF_2_NH + F	129	18	5.2 × 10^−32^
NF_3_ + N	NF_2_ + NF	138	−70	8.0 × 10^−32^
NF_3_ + N	NF_2_N + F	167	−122	4.4 × 10^−36^
NHF_2_ + H	NHF + HF	64	−294	1.6 × 10^−19^
NHF_2_ + H	NH_2_F + F	45	−102	3.2 × 10^−17^
NHF_2_ + H	NF_2_ + H_2_	17	−125	2.6 × 10^−13^
NH_2_F + H	NH_2_ + HF	58	−281	5.8 × 10^−19^
NH_2_F + H	NH_3_ + F	17	−157	1.1 × 10^−13^
NH_2_F + H	NHF + H_2_	35	−58	3.5 × 10^−15^

The initial reaction taking place in the etching chamber is considered to be NF_3_ + H, which produces NF_2_, HF, NHF_2_, and F. The F atoms rapidly react with NH_3_ in the chamber with the hydrogen abstraction mechanism as:

R3 NH_3_ + F → NH_2_ + HF

with the rate constants on the order of 10^−11^ cm^3^ per molecule per s.^32^ Subsequent reactions of the other products, NHF_2_ and NF_2_, and their hydrogen-substituted products, NH_2_F and NHF, are investigated below.

The reactions of NHF_2_ and NH_2_F with H atoms each have three reaction channels—fluorine abstraction, F/H substitution, and hydrogen abstraction:

R4a NHF_2_ + H → NHF + HF

R4b NHF_2_ + H → NH_2_F + F

R4c NHF_2_ + H → NF_2_ + H_2_

R5a NH_2_F + H → NH_2_ + HF

R5b NH_2_F + H → NH_3_ + F

R5c NH_2_F + H → NHF + H_2_

The barrier heights and reaction energies for these channels are listed in Table 1. The NHF_2_ + H reaction dominantly occurs with the hydrogen abstraction channel (R4c), with a calculated barrier height of 17 kJ mol^−1^ and rate constant of 2.6 × 10^−13^ cm^3^ per molecule per s at 350 K. The other two channels have rate constants that are several orders of magnitude smaller due to their higher barrier heights. In contrast, the reaction of NH_2_F with H atoms is dominated by the substitution channel (R5b), with a barrier height (17 kJ mol^−1^) and rate constant (1.1 × 10^−13^ cm^3^ per molecule per s at 350 K) comparable with those of (R4c). The hydrogen abstraction channel is less favorable for the NH_2_F + H reaction; its rate constant is more than an order of magnitude smaller than that for the substitution channel. The different preferences of the reaction channels in the NHF_2_ + H and NH_2_F + H reactions might be explained by the large electronegativity of fluorine atoms, which makes the NF_2_ radicals relatively stable compared to the NH_2_ and NHF radicals.

**Fig. 4:**
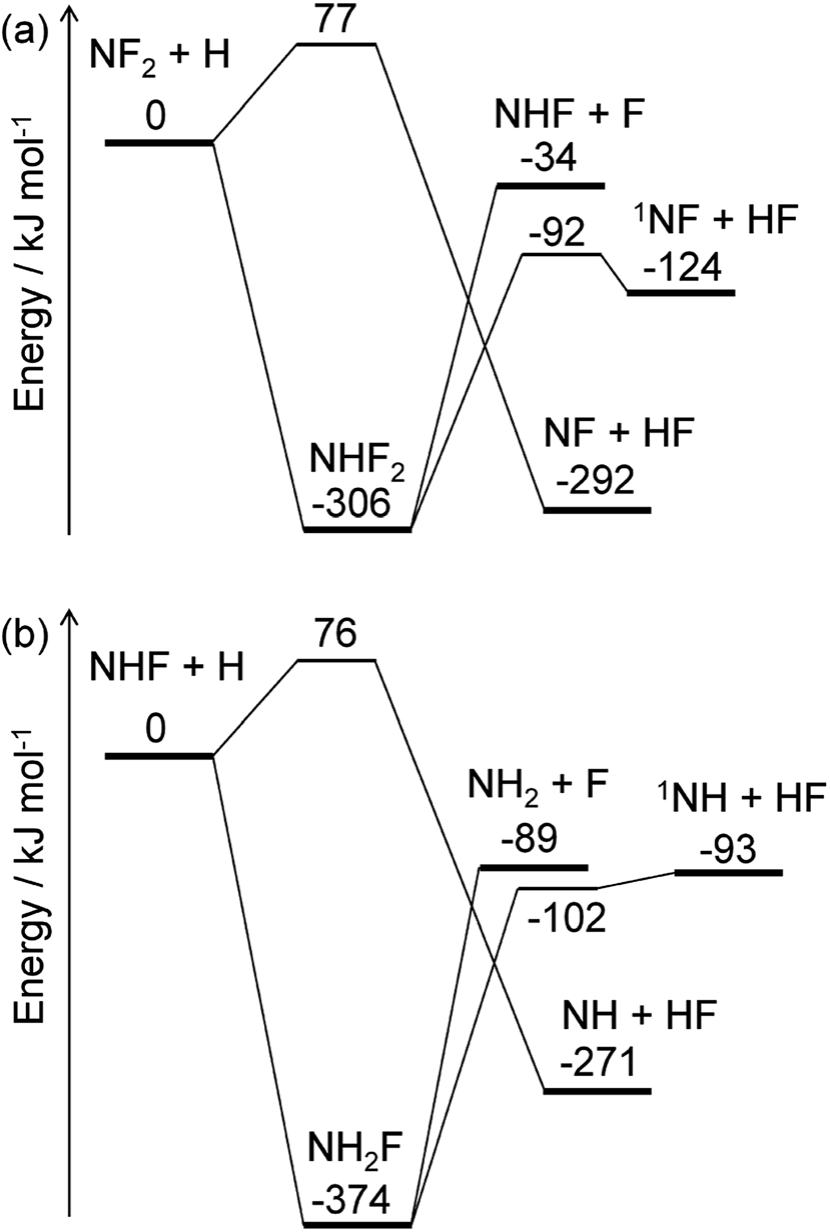
Energy diagrams for the (a) NF_2_ + H and (b) NHF + H reactions. The ZPE-corrected ground-state energies relative to the reactants are shown.

The energy diagrams for the NF_2_ + H and NHF + H reactions are shown in Fig. 4. Both reactions have fluorine abstraction and recombination pathways. The fluorine abstractions occur on triplet potential energy surfaces and produce triplet species, NF and NH. Their high barrier heights give small rate constants, ∼10^−21^ cm^3^ per molecule per s at 350 K, for the fluorine abstraction channels. On the other hand, the recombination channels have barrierless potential energy surfaces for the association of the radicals. The formed NHF_2_ and NH_2_F molecules are chemically activated and either stabilize by collisions with third-body molecules or dissociate to fragments. Both NHF_2_ and NH_2_F intermediates have two dissociation channels: barrierless N–F bond fission and concerted HF elimination. The dissociation channels lie lower in energy than the reactants; therefore, the activated intermediates are expected to directly dissociate to the fragments. For the master equation calculations, the microscopic rate constants for the barrierless channels were calculated using the inverse Laplace transform^19^ of the high-pressure limiting rate constants. The rate constants for NF_2_ + H and NHF + F were reported to be ∼4 × 10^−11^ and ∼2 × 10^−10^ cm^3^ per molecule per s, respectively.^33,34^ These values are assumed to be in the high-pressure limits. The same values are also used for the NHF + H and NH_2_ + F reactions, respectively. The microscopic rate constants for the dissociation channels were calculated from these values and the calculated equilibrium constants. For the collisional energy transfer processes, several models of collision frequency and energy transfer were tested, but the resultant rate constants were found to be insensitive to the models and their parameters at the pressure of 200 Pa. Therefore, the Lennard-Jones collision frequencies and the exponential down model^18^ were employed here, with the Lennard-Jones parameters of *σ* = 4 Å and *ε* = 200 cm^−1^ and the energy transfer parameter of 〈Δ*E*_down_〉 = 0.6 (*T*/K) cm^−1^. The calculated rate constants showed the dominance of the N–F bond fission and HF elimination channels:

R6a NF_2_ + H → NHF + F

R6b NF_2_ + H → ^1^NF + HF

R7a NHF + H → NH_2_ + F

R7b NHF + H → ^1^NH + HF

with the branching fractions of 0.78 and 0.22 for (R6a) and (R6b), respectively, and 0.998 and 0.002 for (R7a) and (R7b), respectively. These results suggest that NF_2_ radicals produced in the initial reaction (R2a) mainly produce NHF radicals by (R6a), which subsequently yield NH_2_ radicals by (R7a). The F atoms generated in each reaction are consumed by the reaction (R3). The ^1^NF produced in the minor channel, (R6b), may react with NH_3_; analogously to the ^1^NH + NH_3_ reaction,^35^ the following reaction

R8 ^1^NF + NH_3_ → NHF + NH_2_

was assumed to occur with the rate constant of 1.5 × 10^−10^ cm^3^ per molecule per s.

### 3.2 Kinetic simulation and reaction pathways

The kinetic model describing the gas-phase reactions has been constructed based on the rate constants described above and other reactions implemented in kinetic models for combustion of nitrogen-containing species.^6–8^ The calculated rate constants were represented by the modified Arrhenius expression, the parameters of which were determined by least-squares fittings over the temperature range of 250–1000 K. The reactions involving H and N elements were adopted from the models of Coppens *et al.*^6^ and Glarborg *et al.*;^7^ although their models were developed to model NO_*x*_ formation and NH_3_ oxidation in combustion, they have detailed descriptions of the reactions of nitrogen hydrides that are applicable to the present purpose. The reactions involving fluorine and nitrogen fluorides were taken from the model developed for H_2_/NF_3_ flames.^8^ The constructed model consists of 25 species and 131 reactions and is available in the ESI.†

**Fig. 5:**
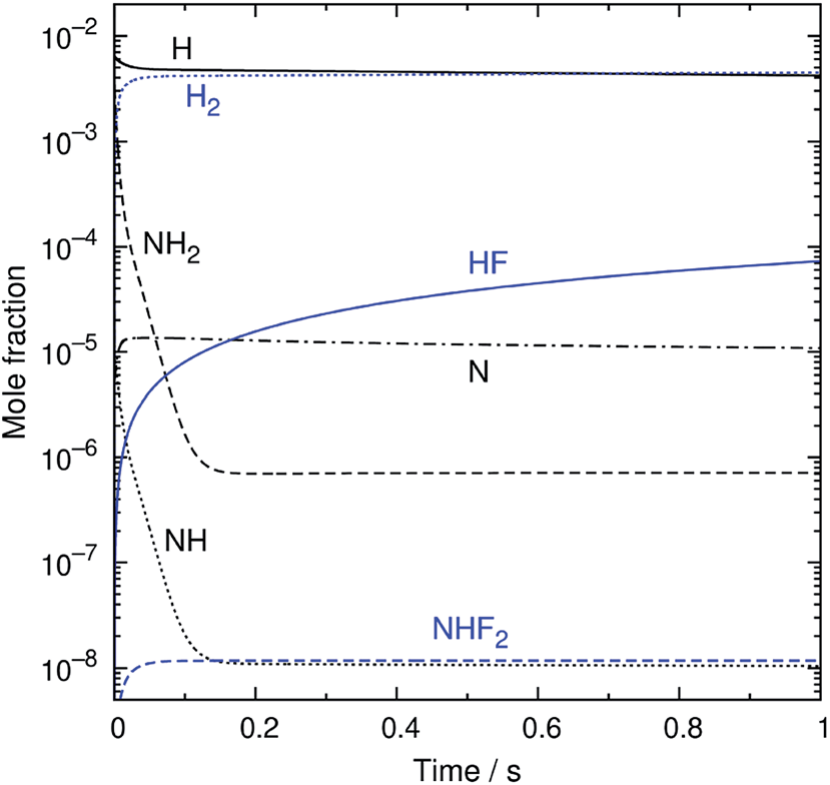
The simulated time profiles of the major reaction intermediates and products.

An example of the simulated profiles of major intermediates and products is shown in Fig. 5 as a function of time (*t*). The kinetic simulation was performed for the representative operation condition previously mentioned. The concentrations of the supplied gases (NF_3_, NH_3_, and N_2_) were virtually unchanged from the initial condition and, therefore, are not plotted here. The radical species produced from the discharge rapidly reacted during the early stage of the simulation, and their temporal profiles became nearly steady within the first 150 ms. After that, there were slight gradual decreases of the radical concentrations accompanied by the formation of HF. The partial pressures of H atoms and HF were about 1 and 0.02 Pa at *t* = 1 s. These species, as well as NH_3_, are considered to contribute to etching, as discussed later.

**Fig. 6:**
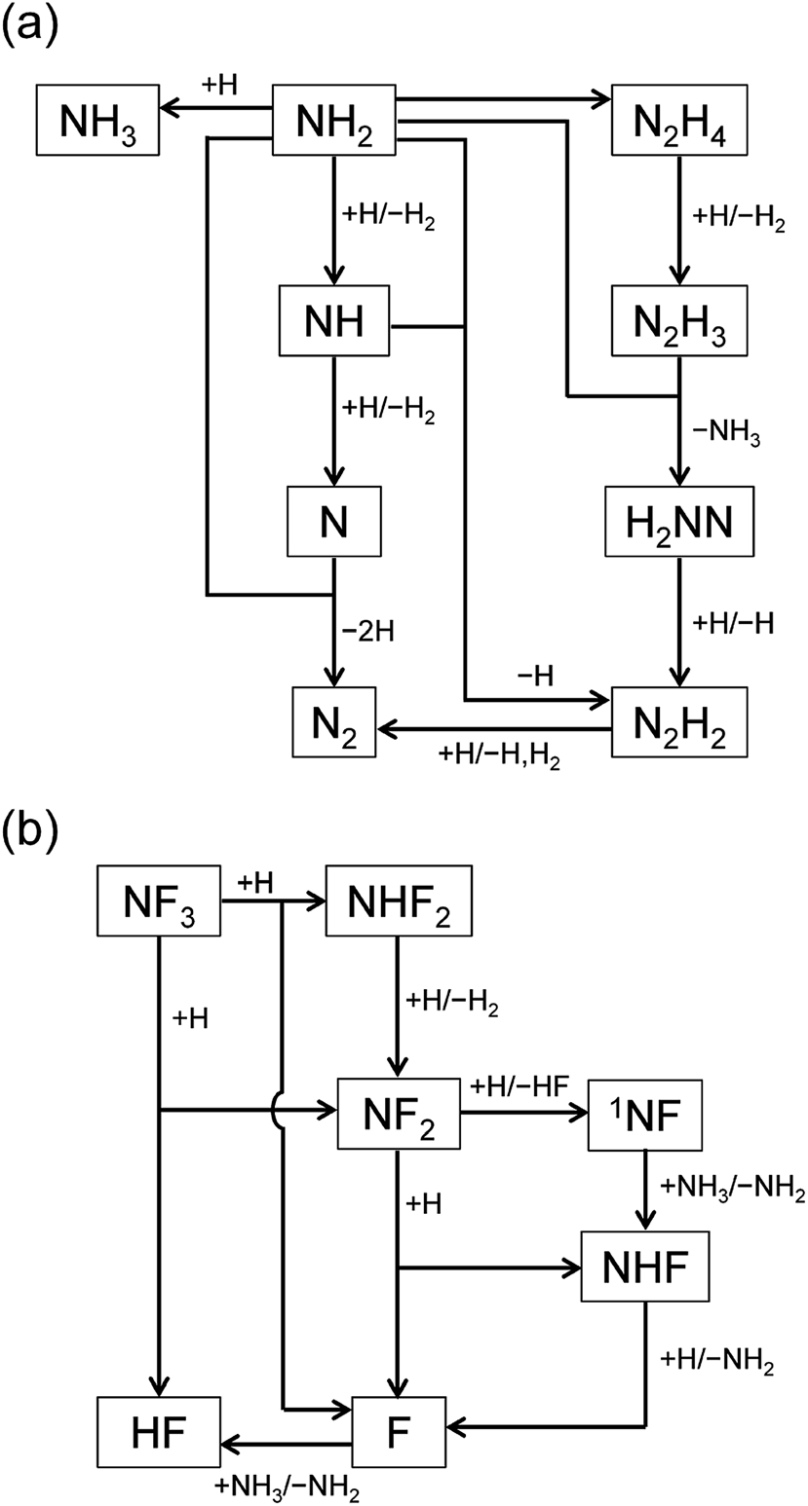
Schematic diagrams of the major reaction pathways at (a) *t* = 1 ms and (b) *t* = 1 s.

The reaction pathways were analyzed by the mass flux analysis.^36^ The major reaction pathways are schematically depicted in Fig. 6. At the early stage, NH_2_, NH, and N radicals are consumed by the following reactions to generate H_2_ and N_2_:

R9 NH_2_ + H → NH + H_2_

R10 NH + H → N + H_2_

R11 N + NH_2_ → 2H + N_2_

This sequence of reactions can be viewed as the H-atom catalyzed conversion of 2NH_2_ into 2H_2_ + N_2_, in which the H atoms are consumed by the reactions with NH_2_ and NH but are generated by the N + NH_2_ reaction, resulting in a steady H-atom concentration. The other reactions contributing to the radical concentration profiles include the formation of NH_3_ and N_2_H_4_ by the recombination reactions of NH_2_ + H (R12) and NH_2_ + NH_2_(R13), respectively, the formation of N_2_H_2_ from the reaction between NH_2_ and NH (R14), and the reactions of N_2_H_*x*_ (*x* = 2–4) species that finally results in the formation of N_2_(R15)–(R18):

R12 NH_2_ + H + M → NH_3_ + M

R13 NH_2_ + NH_2_ + M → N_2_H_4_ + M

R14 NH_2_ + NH → N_2_H_2_ + H

R15 N_2_H_4_ + H → N_2_H_3_ + H_2_

R16 N_2_H_3_ + NH_2_ → H_2_NN + NH_3_

R17 H_2_NN + H → N_2_H_2_ + H

R18 N_2_H_2_ + H → N_2_ + H_2_ + H

where M represents third-body molecules.

The reaction pathways involving fluorine-containing species are illustrated in Fig. 6(b). The reaction sequence is initiated by the NF_3_ + H reaction, which dominantly generates NF_2_ radicals and HF. As a minor product, NHF_2_ can also be formed in a small amount, but is readily converted to NF_2_ radicals by the H abstraction reaction (R4c). The NF_2_ radicals have two channels in their reactions with H atoms, (R6a) and (R6b), producing NHF + F and ^1^NF + HF, respectively. The ^1^NF radicals generated in the latter channel produce NHF radicals by their reaction with NH_3_(R8); therefore, the two channels in the NF_2_ + H reaction practically lead to the same products. The NHF radicals then react with H atoms to form NH_2_ + F, and F atoms produced in this and other reactions generate HF *via* their reaction with NH_3_(R3).

The overall reaction process may be roughly represented as NF_3_ + 2NH_3_ + 3H → 3NH_2_ + 3HF. If the conversion of NH_2_ is also considered, this translates into NF_3_ + 2NH_3_ + 3H → (3/2)N_2_ + 3H_2_ + 3HF. The rate-limiting step is the initial reaction of NF_3_ with H atoms, but the secondary and side reactions also contribute to the temporal behavior of the reaction intermediates and products. The simulated time profiles, except for the early behavior of the NH_2_ and NH radicals, can be approximately described by a simplified model composed of 12 reactions listed in Table 2 (also available in the ESI†). This simplified model should be useful for more realistic and detailed simulations of the etching process, which include fluid dynamics and surface reactions.

**Table 2:** Reactions and their rate constants^a^ of the simplified kinetic model

Reaction	*A* b	*n*	*E*_a_/*R*^c^	Reference
NF_3_ + H ⇄ NF_2_ + HF	5.0 × 10^−27^	5.188	4228	This work
NF_3_ + H ⇄ NHF_2_ + F	1.1 × 10^−32^	6.735	4090	This work
NHF_2_ + H ⇄ NF_2_ + H_2_	2.8 × 10^−19^	2.628	563.9	This work
NF_2_ + H ⇄ NHF + F	1.3 × 10^−11^	0.132	−34.8	This work
NF_2_ + H ⇄ ^1^NF + HF	5.4 × 10^−10^	−0.616	177.5	This work
^1^NF + NH_3_ ⇄ NHF + NH_2_	1.5 × 10^−10^	0	0	See text
NHF + H ⇄ NH_2_ + F	4.0 × 10^−11^	0	0.05	This work
NH_3_ + F ⇄ NH_2_ + HF	2.5 × 10^−10^	0	650.2	32
NH + H_2_ ⇄ NH_2_ + H	3.5 × 10^−11^	0	7758	7
NH + H ⇄ N + H_2_	5.3 × 10^−11^	0	163.5	6
NH_2_ + N ⇄ N_2_ + H + H	1.1 × 10^−10^	0	0	6
H + H + M ⇄ H_2_ + M	1.5 × 10^−29^	−1.3	0	6

a Parameters for the modified Arrhenius expression, *k* = *A* (*T*/K)^*n*^ exp(−*E*_a_/*RT*), are given.

b In units of cm^3^ per molecule per s and cm^6^ per molecule^2^ per s for bimolecular and termolecular reactions, respectively.

c In units of K.

### 3.3 Implication to surface process

Modeling of the surface reactions will be an important next step for understanding and simulating the etching process. Although the present study primarily focuses on the gas-phase processes, some implications derived from preliminary calculations of ammonium fluoride clusters, (NH_3_)_*n*_(HF)_*m*_, which have been suggested to contribute to the etching of SiO_2_ layers,^1^ are presented here.

**Fig. 7:**
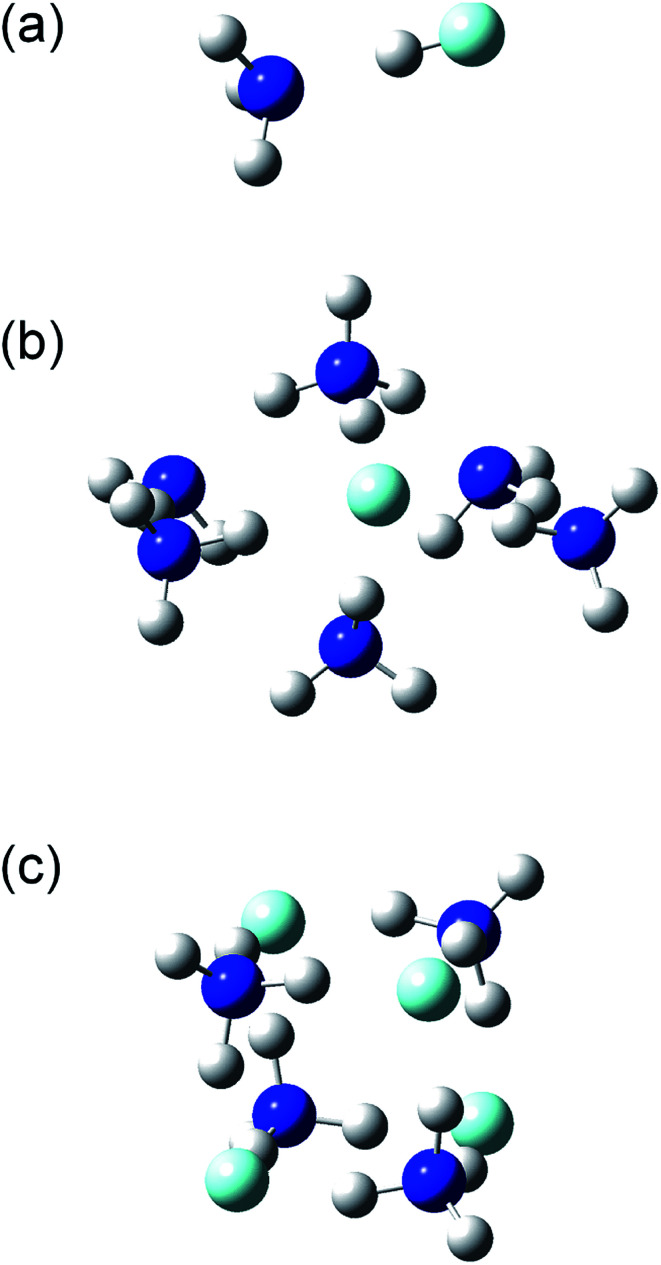
Structures of the (a) (NH_3_)(HF), (b) (NH_3_)_6_(HF), and (c) (NH_3_)_4_(HF)_4_ clusters.

The structures and energies of the (NH_3_)_*n*_(HF)_*m*_ clusters with *m* = 1 and *n* = 1–7, 11, and 13, as well as *n* = *m* = 1–6, were calculated. The calculated structures are shown in the ESI,† and a few representative ones, (*n*, *m*) = (1, 1), (6, 1), and (4, 4), are shown in Fig. 7. For each composition, the presented structure corresponds to the isomer that had the lowest potential energy among several local minima calculated at the CBS-QB3//ωB97X-D level of theory. Since the relative energies for the isomers of large clusters are expected to sensitively depend on the computational method used, these structures may not necessarily be those of the most stable isomers. The small clusters of (*n*, *m*) = (1, 1), (2, 1), (3, 1), and (2, 2) are formed by the dipole–dipole interaction between the NH_3_ and HF molecules. The calculated H–F bond lengths are shorter than 1 Å for the small clusters but increase as the cluster size becomes larger. For example, the (NH_3_)_6_(HF) cluster shown in Fig. 7(b) has an H–F bond length of 1.39 Å, and the H atom is placed rather close to the adjacent NH_3_ molecule, with a N–H bond length of 1.11 Å. The Mulliken charge of the F atom was calculated to be −0.72 at the MP2/CBSB3 level, clearly indicating the ionic nature and that the cluster should rather be denoted as (NH_3_)_5_(NH_4_^+^)(F^−^). Similarly, the cluster shown in Fig. 7(c) has the structure that can be represented as (NH_4_^+^)_4_(F^−^)_4_. The ionic nature is more pronounced in larger clusters; for example, the H–F bond lengths in the (*n*, *m*) = (11, 1), (15, 1), (4, 4), (5, 5), and (6, 6) clusters are in the range 1.59–1.68 Å.

Thermodynamic quantities of the clusters were calculated using the CBS-QB3//ωB97X-D method. Table 3 lists the calculated enthalpy (Δ*H*°), entropy (Δ*S*°), and Gibbs energy (Δ*G*°) changes in the (NH_3_)_*n*_(HF)_*m*_ cluster formation from isolated molecules, *n*NH_3_ + *m*HF, at the standard state (298 K, 1 bar). For the (NH_3_)_*n*_(HF) clusters, the standard enthalpy and entropy changes decrease by approximately 25 kJ mol^−1^ and 120 J K^−1^ mol^−1^, respectively, for each increment of *n*, which results in a monotonic increase of the standard Gibbs energy change as the cluster size increases. Although the rigid-rotor and harmonic-oscillator approximations possibly underestimate the entropies of large clusters, the magnitude of the entropy loss is considered reasonable because it is comparable with the reduction of the translational entropy in solvation.^37^ The entropies of the (NH_3_)_*n*_(HF)_*n*_ clusters show a similar trend with respect to the cluster size, but their enthalpies have a steeper dependence on the cluster size than those of the (NH_3_)_*n*_(HF) clusters. As a result, the standard Gibbs energy changes in the formation of the (NH_3_)_*n*_(HF)_*n*_ clusters decrease as the cluster size increases. This indicates homogeneous nucleation of ammonium fluoride clusters under the standard state condition. On the other hand, the Gibbs energy changes at 200 Pa listed in Table 3 all have positive values and become increasingly larger for larger clusters; therefore, no cluster formation is expected in the gas phase under typical operation conditions of the etching process.

**Table 3:** Calculated enthalpies, entropies, and Gibbs energies of the (NH_3_)_*n*_(HF)_*m*_ clusters relative to *n*NH_3_ + *m*HF

*n*, *m*	Δ*H* (298 K)/kJ mol^−1^	Δ*S* (298 K)/J K^−1^ mol^−1^	Δ*G* (298 K)/kJ mol^−1^	Δ*G* (298 K, 200 Pa)/kJ mol^−1^	Δ*G* (350 K, 200 Pa)/kJ mol^−1^
1, 1	−46	−112	−12	3	12
2, 1	−69	−219	−3	28	44
3, 1	−95	−342	7	43	79
4, 1	−115	−447	19	80	114
5, 1	−134	−597	44	121	165
6, 1	−158	−743	64	156	211
7, 1	−193	−856	62	170	233
11, 1	−290	−1407	129	299	401
15, 1	−369	−1912	201	432	571
2, 2	−137	−361	−29	17	44
3, 3	−218	−631	−30	47	93
4, 4	−345	−1002	−46	62	133
5, 5	−448	−1230	−81	57	145
6, 6	−567	−1555	−104	66	176

**Fig. 8:**
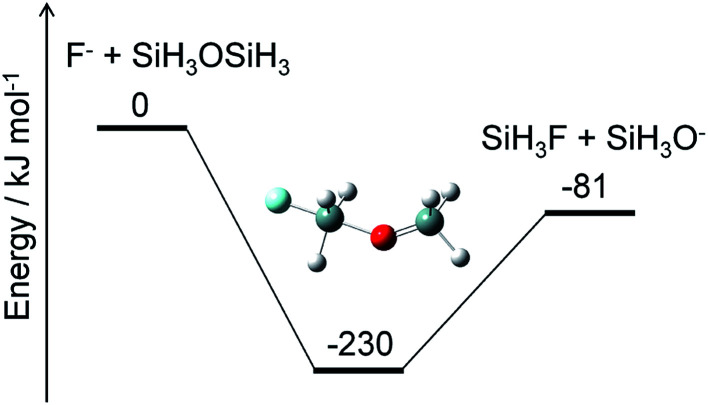
Energy diagram for a prototype etching reaction and structure of the intermediate. The ZPE-corrected ground-state energies relative to the reactants are shown.

Alternatively, ammonium fluoride clusters can potentially be formed on SiO_2_ surfaces because of the electrostatic interaction between them. The polar NH_3_ and HF molecules adsorbed onto the surface can interact with each other as well as with the partially charged O and Si atoms in the SiO_2_ layer. The existence of the SiO_2_ surface should facilitate cluster formation and charge separation in the cluster. Then, the negatively charged F atoms in the cluster can attack the positively charged Si atoms in the SiO_2_ layer and break a Si–O bond by a nucleophilic substitution reaction. A crude example of this reaction is shown in Fig. 8, where F^−^ and SiH_3_OSiH_3_ (disiloxane) serve as prototypes of negatively charged F atoms in the cluster and a Si–O bond, respectively. The nucleophilic substitution is exothermic and can proceed without any energy barrier. A similar mechanism is expected to take place between the ammonium fluoride clusters and the SiO_2_ surface; a negatively charged F atom in the cluster substitutes the Si–O–Si structure in the SiO_2_ layer with a Si–F bond, leaving a Si–O site on the surface. Hydrogen atoms can attach to the Si–O site to form a hydroxy group, Si–OH, which can further react with the F and H atoms to produce another Si–F bond and H_2_O. This cycle can be repeated as long as the F and H atoms are supplied to destruct the SiO_2_ layers. The volatile products, such as H_2_O and SiF_4_, are removed from the surface, but some silicon fluoride products remain on the surface to form (NH_4_)_2_SiF_6_.^1,5^ Modeling of these processes requires elucidation of the thermodynamics and kinetics of adsorption, desorption, and reactions on the surface.

## 4. Conclusion

To summarize the results of the rate constant calculations and kinetic simulation, the following reactions are found to primarily contribute to the gas-phase process in the etching chamber:

R2a NF_3_ + H → NF_2_ + HF

R6a NF_2_ + H → NHF + F

R6b NF_2_ + H → ^1^NF + HF

R8 ^1^NF + NH_3_ → NHF + NH_2_

R7a NHF + H → NH_2_ + F

R3 NH_3_ + H → NH_2_ + HF

where the NF_3_ + H reaction (R2a) is the rate-limiting step. The whole reactions may be summarized by the overall reaction NF_3_ + 2NH_3_ + 3H → (3/2)N_2_ + 3H_2_ + 3HF. Detailed and simplified reaction models were constructed that can be used to predict the species concentrations and spatial distribution in the chamber when combined with fluid dynamics calculations. Future studies in this line would include investigation of the spatial uniformity of the etching rate and its dependence on process parameters.

The preliminary calculation of the ammonium fluoride clusters suggested the formation of negatively charged F atoms in the cluster, which can destruct the SiO_2_ layer by successive nucleophilic substitution reactions. Therefore, the etching process is suggested to comprise the following steps: generation of etchant species from the gas-phase reactions, adsorption (cluster formation) and desorption of the etchant species on the surface, and surface reactions by the negatively charged F atoms.

## Conflicts of interest

This study is supported by ULVAC, Inc.

## Supplementary Material

RA-010-D0RA05726F-s001
