# Supplementary material for: Gas-phase reaction mechanism in chemical dry etching using NF3 and remotely discharged NH3/N2 mixture
Source: RSC Adv. 2020 Aug 20;10(51):30806–14. doi: 10.1039/d0ra05726f (PMC9056326; doi:10.1039/d0ra05726f)
Supplement: RA-010-D0RA05726F-s001 [file RA-010-D0RA05726F-s001.pdf]

Electronic Supplementary Material (ESI) for RSC Advances.
This journal is © The Royal Society of Chemistry 2020

Supplementary Material for "Gas-phase reaction mechanism in chemical dry etching using NF_3 and remotely discharged NH_3/N_2 mixture"

Kinetic model (Chemkin format)

```
ELEMENTS
 H N F
END
SPECIES
 H H2 N NH NH2 NH3 H2NN N2 NNH N2H2 N2H3 N2H4
 F F2 HF NF3 NF2 NF NHF NHF2 NH2F N2F2 N2F4 NFsing NHsing
END
THERMO ALL
   300.000  1000.000  5000.000
H                 L 6/94H   1             G   200.000  6000.00  1000.0      1
 0.25000000E+01 0.00000000E+00 0.00000000E+00 0.00000000E+00 0.00000000E+00    2
 0.25473660E+05-0.44668285E+00 0.25000000E+01 0.00000000E+00 0.00000000E+00    3
 0.00000000E+00 0.00000000E+00 0.25473660E+05-0.44668285E+00                   4
H2  REF ELEMENT   RUS 78H   2             G   200.000  6000.00  1000.0      1
 0.29328305E+01 0.82659802E-03-0.14640057E-06 0.15409851E-10-0.68879615E-15    2
-0.81305582E+03-0.10243164E+01 0.23443029E+01 0.79804248E-02-0.19477917E-04    3
 0.20156967E-07-0.73760289E-11-0.91792413E+03 0.68300218E+00                   4
N                 L 6/88N   1             G   200.000  6000.00  1000.0      1
 0.24159429E+01 0.17489065E-03-0.11902369E-06 0.30226244E-10-0.20360983E-14    2
 0.56133775E+05 0.46496095E+01 0.25000000E+01 0.00000000E+00 0.00000000E+00    3
 0.00000000E+00 0.00000000E+00 0.56104638E+05 0.41939088E+01                   4
NH                L11/89N   1H   1        G   200.000  6000.00  1000.0      1
 0.27836929E+01 0.13298429E-02-0.42478047E-06 0.78348504E-10-0.55044470E-14    2
 0.42134514E+05 0.57407798E+01 0.34929084E+01 0.31179197E-03-0.14890484E-05    3
 0.24816442E-08-0.10356967E-11 0.41894294E+05 0.18483277E+01                   4
NH2               L12/89N   1H   2        G   200.000  6000.00  1000.0      1
 0.28476611E+01 0.31428453E-02-0.89866557E-06 0.13032357E-09-0.74885356E-14    2
 0.21823916E+05 0.64718133E+01 0.42055601E+01-0.21355282E-02 0.72682021E-05    3
-0.59302799E-08 0.18067218E-11 0.21535223E+05-0.14663231E+00                   4
NH3  AMONIA       RUS 89N   1H   3        G   200.000  6000.00  1000.0      1
 2.71709692E+00 5.56856338E-03-1.76886396E-06 2.67417260E-10-1.52731419E-14    2
-6.58451989E+03 6.09289837E+00 4.30177808E+00-4.77127330E-03 2.19341619E-05    3
-2.29856489E-08 8.28992268E-12-6.74806394E+03-6.90644393E-01                   4
N2  REF ELEMENT   RUS 78N   2             G   200.000  6000.00  1000.0      1
 0.29525407E+01 0.13968838E-02-0.49262577E-06 0.78600091E-10-0.46074978E-14    2
-0.92393753E+03 0.58718221E+01 0.35309628E+01-0.12365950E-03-0.50299339E-06    3
 0.24352768E-08-0.14087954E-11-0.10469637E+04 0.29674391E+01                   4
NNH               T07/93N   2H   1        G   200.000  6000.00  1000.0      1
 0.37667545E+01 0.28915081E-02-0.10416620E-05 0.16842594E-09-0.10091896E-13    2
 0.28650697E+05 0.44705068E+01 0.43446927E+01-0.48497072E-02 0.20059459E-04    3
-0.21726464E-07 0.79469538E-11 0.28791973E+05 0.29779411E+01                   4
N2H2              L 5/90N   2H   2        G   200.000  6000.00  1000.0      1
 0.13111509E+01 0.90018727E-02-0.31491187E-05 0.48144969E-09-0.27189798E-13    2
 0.24786417E+05 0.16409109E+02 0.49106602E+01-0.10779187E-01 0.38651644E-04    3
-0.38650163E-07 0.13485210E-10 0.24224273E+05 0.91027970E-01                   4
```

```
N2H3              T 7/93H   3N   2          G   200.000  6000.00  1000.0       1
 0.43414654E+01 0.75280979E-02-0.27478351E-05 0.44688178E-09-0.26846990E-13    2
 0.25176779E+05 0.98835045E+00 0.33151120E+01 0.21514763E-02 0.21849694E-04    3
-0.29813376E-07 0.12038856E-10 0.25844190E+05 0.82263324E+01                   4
N2H4 HYDRAZINE    L 5/90N   2H   4          G   200.000  6000.00  1000.0       1
 4.93957357E+00 8.75017187E-03-2.99399058E-06 4.67278418E-10-2.73068599E-14    2
 9.28265548E+03-2.69439772E+00 3.83472149E+00-6.49129555E-04 3.76848463E-05    3
-5.00709182E-08 2.03362064E-11 1.00893925E+04 5.75272030E+00                   4
H2NN Isodiazene   T 9/11N  2.H  2.   0.    0.G   200.000  6000.000 1000.       1
 3.05903670E+00 6.18382347E-03-2.22171165E-06 3.58539206E-10-2.14532905E-14    2
 3.48530149E+04 6.69893515E+00 4.53204001E+00-7.32418578E-03 3.00803713E-05    3
-3.04000551E-08 1.04700639E-11 3.49580003E+04 1.51074195E+00 3.61943157E+04    4
F                 thLi97F   1               G   200.00   5000.00  1000.00      1
 2.68472005E+00-2.01550623E-04 8.73230990E-08-1.68631530E-11 1.20225312E-15    2
 8.78162871E+03 3.90029730E+00 2.41887090E+00 2.94592975E-03-8.93797264E-06    3
 9.92947105E-09-3.80146744E-12 8.75611997E+03 4.73710830E+00                   4
F2                thLi97F   2               G   200.00   5000.00  1000.00      1
 4.14815398E+00 2.20610581E-04 1.84710927E-07-1.02699999E-10 1.08022713E-14    2
-1.33548505E+03 4.62571682E-01 3.20838067E+00 1.25799380E-03 3.90234858E-06    3
-7.22898588E-09 3.32184059E-12-1.03425209E+03 5.60576275E+00                   4
HF                thLi97H   1F   1          G   200.00   5000.00  1000.00      1
 3.02051188E+00 6.59427921E-04-3.46581819E-08-1.90227207E-11 2.57439346E-15    2
-3.37216641E+04 3.65476705E+00 3.48099578E+00 2.15604711E-04-6.98126549E-07    3
 8.70863939E-10-2.40504593E-13-3.39129362E+04 1.01355519E+00                   4
NF3               thLi97N   1F   3          G   200.00   5000.00  1000.00      1
 7.82521172E+00 2.73753383E-03-1.09529268E-06 2.22364403E-10-1.60848883E-14    2
-1.86685131E+04-1.49077998E+01 1.31904512E+00 2.34281290E-02-2.35120746E-05    3
 8.24365283E-09 1.94980036E-13-1.70829185E+04 1.78264736E+01                   4
NF2               thLi97N   1F   2          G   200.00   5000.00  1000.00      1
 5.67641174E+00 1.53448742E-03-6.75630227E-07 1.33193899E-10-9.63671685E-15    2
 2.16476624E+03-3.27595375E+00 3.03793228E+00 6.63330462E-03 1.60571306E-06    3
-9.87535658E-09 5.25723274E-12 2.94215307E+03 1.07299940E+01                   4
NF                thLi97N   1F   1          G   200.00   5000.00  1000.00      1
 3.86263825E+00 7.43974747E-04-3.24630959E-07 7.98716895E-11-7.26947655E-15    2
 2.67420491E+04 3.16825533E+00 3.59936626E+00-2.18238902E-03 1.14120083E-05    3
-1.40082209E-08 5.53381956E-12 2.69700952E+04 5.35567652E+00                   4
NHF               thLi97N   1H   1F   1     G   200.00   5000.00  1000.00      1
 3.71158561E+00 3.06387172E-03-1.20211924E-06 2.17526300E-10-1.46590295E-14    2
 1.21636321E+04 5.57080381E+00 4.14778046E+00-3.33523020E-03 1.76254768E-05    3
-2.05636974E-08 7.90187565E-12 1.22631218E+04 4.49092134E+00                   4
NHF2              thLi97N   1H   1F   2     G   200.00   5000.00  1000.00      1
 5.28016518E+00 4.67485094E-03-1.91039694E-06 3.55656720E-10-2.47536606E-14    2
-1.44295582E+04-1.63935123E+00 3.32084065E+00 3.50799621E-03 1.62618893E-05    3
-2.57045784E-08 1.09893746E-11-1.36320019E+04 9.90910778E+00                   4
NH2F              thLi97N   1H   2F   1     G   200.00   5000.00  1000.00      1
 3.01480038E+00 6.47562008E-03-2.52146299E-06 4.53309231E-10-3.07557485E-14    2
-1.03025226E+04 8.34023263E+00 4.43116859E+00-7.00799603E-03 3.24393526E-05    3
-3.55353926E-08 1.30643784E-11-1.02525393E+04 3.28196636E+00                   4
N2F2              thLi97N   2F   2          G   200.00   5000.00  1000.00      1
 7.63877339E+00 2.67149446E-03-1.18679576E-06 2.33362669E-10-1.68604530E-14    2
 4.82463668E+03-1.27058184E+01 2.69382300E+00 2.00013286E-02-2.52532378E-05    3
 1.59830194E-08-4.08495887E-12 6.00302286E+03 1.19232147E+01                   4
N2F4              thLi97N   2F   4          G   200.00   5000.00  1000.00      1
```

```
 1.28420508E+01 3.65302482E-03-1.64451502E-06 3.26122901E-10-2.36984310E-14    2
-7.17565227E+03-3.73283563E+01 1.33555669E+00 4.73944673E-02-6.67860771E-05    3
 4.50611605E-08-1.18521223E-11-4.64409997E+03 1.90304547E+01                   4
NHsing            ATcT3EH   1N   1    0    0G    200.00   6000.00 1000.00      1
 2.87862282E+00 1.12796710E-03-2.61325432E-07 3.22484343E-11-1.52587969E-15    2
 6.04227321E+04 4.83507631E+00 3.45989150E+00 4.96390086E-04-1.94274205E-06    3
 3.02845800E-09-1.26601050E-12 6.02215686E+04 1.60948444E+00 6.12634941E+04    4
NFsing             cbswbN   1F   1          G   200.000  5000.000 1080.00      1
 3.83021305E+00 7.18946353E-04-3.06028586E-07 5.82803458E-11-4.11804265E-15    2
 4.65119761E+04 2.22170527E+00 3.43724845E+00-6.95919156E-04 6.43252150E-06    3
-7.74659319E-09 2.85740270E-12 4.67456918E+04 4.86861465E+00                   4
END
REACTIONS
H+H+M=H2+M                    7.0E+17     -1.000          0. ! Coppens2007CNF
          H2/0/ N2/0/ H/0/
H+H+H2=H2+H2                  1.0E+17     -0.600          0. ! Coppens2007CNF
H+H+N2=H2+N2                  5.4E+18     -1.300          0. ! Coppens2007CNF
H+H+H=H2+H                    3.2E+15      0.000          0. ! Coppens2007CNF
N2+M=N+N+M                    1.0E+28     -3.330     225000. ! Coppens2007CNF
          N/5/
NH+M=N+H+M                   2.65E+14      0.000      75500. ! Coppens2007CNF
NH+H=N+H2                     3.2E+13      0.000        325. ! Coppens2007CNF
NH+N=N2+H                     9.0E+11      0.500          0. ! Coppens2007CNF
NH+NH=NNH+H                   5.1E+13      0.000          0. ! Coppens2007CNF
NH+NH=NH2+N                   5.7E-01      3.880        342. ! Glarborg2018PECS
NH+NH=N2+H2                   1.0E+08      1.000          0. ! Coppens2007CNF
NH2+M=NH+H+M                 3.16E+23     -2.000      91400. ! Coppens2007CNF
NH+H2=NH2+H                   2.1E+13      0.000      15417. ! Glarborg2018PECS
NH2+N=N2+H+H                  6.9E+13      0.000          0. ! Coppens2007CNF
NH2+NH=N2H2+H                 4.3E+14     -0.272        -77. ! Glarborg2018PECS
NH2+NH=NH3+N                  9.6E+03      2.460        107. ! Glarborg2018PECS
NH2+NH2=NH3+NH                5.6E+00      3.530        552. ! Glarborg2018PECS
NH2+NH2=N2H2+H2               1.7E+08      1.620      11783. ! Glarborg2018PECS
NH2+NH2=H2NN+H2               7.2E+04      1.880       8802. ! Glarborg2018PECS
N2H3+H=NH2+NH2                5.0E+13      0.000       2000. ! Coppens2007CNF
NH2+H(+M)=NH3(+M)             1.6E+14      0.000          0. ! Glarborg2018PECS
                  LOW    /    3.6E+22     -1.760          0. /
                  TROE   /      0.5    1E-30     1E30     1E30 /
NH3+M=NH+H2+M                 6.3E+14      0.000      93390. ! Coppens2007CNF
NH3+H=NH2+H2                  6.4E+05      2.390      10171. ! Glarborg2018PECS
NH3+NH2=N2H3+H2               1.0E+11      0.500      21600. ! Coppens2007CNF
NNH=N2+H                      1.0E+09      0.000          0. ! Glarborg2018PECS
NNH+H=N2+H2                   1.0E+14      0.000          0. ! Coppens2007CNF
NNH+N=NH+N2                   3.0E+13      0.000       2000. ! Coppens2007CNF
NNH+NH=N2+NH2                 2.0E+11      0.500       2000. ! Coppens2007CNF
NNH+NH2=N2+NH3                1.0E+13      0.000          0. ! Coppens2007CNF
NNH+NNH=N2H2+N2               1.0E+13      0.000       4000. ! Coppens2007CNF
N2H2+M=NNH+H+M                1.9E+27     -3.050      66107. ! Glarborg2018PECS
N2H2+M=>NH+NH+M              3.16E+16      0.000      99400. ! Coppens2007CNF
          N2/2/ H2/2/
N2H2+H=NNH+H2                 1.1E+14      0.000       3128. ! Glarborg2018PECS
N2H2+N=NNH+NH                 1.0E+06      2.000          0. ! Coppens2007CNF
N2H2+NH=NNH+NH2               2.4E+06      2.000      -1192. ! Glarborg2018PECS
```

```
N2H2+NH2=NH3+NNH              8.8E-02      4.050      -1610. ! Coppens2007CNF
N2H3+NH=N2H2+NH2              2.0E+13      0.000          0. ! Coppens2007CNF
N2H3+NNH=N2H2+N2H2            1.0E+13      0.000       4000. ! Coppens2007CNF
N2H3+M=>NH2+NH+M              5.0E+16      0.000      60000. ! Coppens2007CNF
N2H3+M=>N2H2+H+M              1.0E+16      0.000      37000. ! Coppens2007CNF
N2H3+H=N2H2+H2                2.4E+08      1.500        -10. ! Glarborg2018PECS
N2H3+H=NH+NH3                 1.0E+11      0.000          0. ! Coppens2007CNF
N2H3+N=N2H2+NH                1.0E+06      2.000          0. ! Coppens2007CNF
N2H3+NH2=N2H2+NH3             9.2E+05      1.940      -1152. ! Glarborg2018PECS
N2H3+NH2=H2NN+NH3             3.0E+13      0.000          0. ! Glarborg2018PECS
N2H3+N2H2=N2H4+NNH            1.0E+13      0.000       6000. ! Coppens2007CNF
N2H3+N2H3=NH3+NH3+N2          3.0E+12      0.000          0. ! Coppens2007CNF
N2H3+N2H3=N2H4+N2H2           1.2E+13      0.000          0. ! Coppens2007CNF
NH2+NH2(+M)=N2H4(+M)          5.6E+14     -0.414         66. ! Glarborg2018PECS
                  LOW    /    1.6E+34     -5.49        1987. / !
                  TROE   /     0.31    1E-30     1E30     1E30 / !
N2H4=H2NN+H2                  1.4E+14      0.000      74911. ! Glarborg2018PECS
N2H4+M=>N2H3+H+M              1.0E+15      0.000      63600. ! Coppens2007CNF
          N2/2.4/ NH3/3/ N2H4/4/
N2H4+H=N2H3+H2                7.0E+12      0.000       2500. ! Coppens2007CNF
N2H4+H=NH3+NH2                2.3E+05      1.420       8202. ! Glarborg2018PECS
N2H4+N=N2H3+NH                1.0E+10      1.000       2000. ! Coppens2007CNF
N2H4+NH=NH2+N2H3              1.0E+09      1.500       2000. ! Coppens2007CNF
N2H4+NH2=N2H3+NH3             7.6E-01      4.000       4048. ! Glarborg2018PECS
H2NN=NNH+H                    3.4E+26     -4.830      46228. ! Glarborg2018PECS
H2NN=N2+H2                    2.5E+14      0.000      52785. ! Glarborg2018PECS
H2NN=N2H2                     1.3E+14      0.000      46931. ! Glarborg2018PECS
H2NN+H=NNH+H2                 4.8E+08      1.500       -894. ! Glarborg2018PECS
H2NN+H=N2H2+H                 7.0E+13      0.000          0. ! Glarborg2018PECS
H2NN+NH2=NNH+NH3              1.8E+06      1.940      -1152. ! Glarborg2018PECS
F2+M=F+F+M                   6.06E+13         0.      33783. ! Matsugi2014CNF
  H2/0.4/ HF/0.4/ N2/0.4/ F2/0.0/
F2+F2=F+F+F2                 9.85E+13         0.      34796. ! Matsugi2014CNF
HF+M=H+F+M                  1.147E+22     -1.677   139083.6 ! Matsugi2014CNF
  H2/0.4/ HF/0.4/ N2/0.4/ F2/1.2/
H+F2=HF+F                    2.90E+09        1.4       1325. ! Matsugi2014CNF
F+H2=HF+H                    2.70E+12        0.5        634. ! Matsugi2014CNF
NF3+M=NF2+F+M                9.44E+31     -4.046    61586.6 ! Matsugi2014CNF
  H2/0.4/ HF/0.4/ N2/0.4/ F2/1.2/
NF2+M=NF+F+M                 1.31E+34     -5.004    71082.1 ! Matsugi2014CNF
  H2/0.4/ HF/0.4/ N2/0.4/ F2/1.2/
NF3+H=NF2+HF                 3.01E-03      5.188     8401.8 ! p.w.
NF3+H=NHF2+F                 6.70E-09      6.735     8127.5 ! p.w.
NHF2+H=NHF+HF                2.85E-05      5.752     8020.6 ! p.w.
NHF2+H=NH2F+F                1.56E+02      3.547     6234.0 ! p.w.
NHF2+H=NF2+H2                1.66E+05      2.628     1120.6 ! p.w.
NH2F+H=NH2+HF                7.41E-05      5.549     6981.6 ! p.w.
NH2F+H=NH3+F                 7.56E+07      1.800     2621.1 ! p.w.
NH2F+H=NHF+H2                2.01E-01      4.479     2143.4 ! p.w.
NH3+F=NH2+HF                 1.50E+14         0.       1292. ! Matsugi2014CNF
NH2F+F=NHF+HF                1.50E+14         0.       1292. ! Matsugi2014CNF
NHF2+F=NF2+HF                1.50E+14         0.       1292. ! Matsugi2014CNF
NH2+F=HF+NH                  1.20E+14         0.          0. ! Matsugi2014CNF
```

```
NH+F=N+HF                   1.20E+14      0.          0.  ! Matsugi2014CNF
NF2+F2=NF3+F                4.80E+12      0.      14407.  ! Matsugi2014CNF
NF2+H2=HF+NHF               1.45E+12      0.      17169.  ! Matsugi2014CNF
NF2+H=NF+HF                 2.78E-09      6.902    9468.7 ! p.w.
NF2+H=NFsing+HF             3.25E+14     -0.616     352.7 ! p.w.
NF2+H=NHF+F                 7.87E+12      0.132     -69.2 ! p.w.
NF2+H=NHF2                  7.23E-12      6.180   34391.9 ! p.w.
NHF+F=NFsing+HF             3.32E+16     -0.913     666.8 ! p.w.
NFsing+N2=NF+N2               4E+11       0.          0.  ! (=NHsing+N2)
NFsing+H2=NHF+H               2E+12       0.          0.  ! (=NHsing+H2)
NFsing+NH3=NHF+NH2            9E+13       0.          0.  ! (=NHsing+NH3)
NFsing+NF3=NF2+NF2            9E+13       0.          0.  ! (=NHsing+NH3)
NF2+NF=N2F2+F               1.30E+07      1.509     -480. ! Matsugi2014CNF
NF+H=HF+N                   1.51E+11      0.          0.  ! Matsugi2014CNF
NF+NF=N2+F+F                2.42E+07      1.991     -810. ! Matsugi2014CNF
NF2+NF2=N2F2+F+F            1.50E+13      0.      25000.  ! Matsugi2014CNF
NF3+N=NF2+NF                1.71E+07      1.97    30043.  ! Matsugi2014CNF
N2F4=NF2+NF2                1.39E+15      0.      19852.  ! Matsugi2014CNF
N2F4+H=HF+N2F2+F            2.47E+08      1.734   10970.  ! Matsugi2014CNF
N2F4+H=NF2+NHF2             2.12E+08      1.766   13790.  ! Matsugi2014CNF
N2F4+F=NF2+NF3              3.83E+08      1.605    6720.  ! Matsugi2014CNF
N2F4=N2F2+F+F               1.00E+15      0.      40000.  ! Matsugi2014CNF
N2F4=N2F2+F2                1.00E+12      0.      25000.  ! Matsugi2014CNF
N2F2+H=HF+N2+F              4.40E+08      1.741    7940.  ! Matsugi2014CNF
N2F2=N2+F+F                 2.55E+14      0.283   27910.  ! Matsugi2014CNF
N2F2=N2+F2                  7.55E+13      0.250   37920.  ! Matsugi2014CNF
NHF+F=NHF2                  1.11E+28     -6.019   29808.4 ! p.w.
NFsing+HF=NHF2              3.61E+14     -2.125    3724.5 ! p.w.
NHF+H=NHsing+HF             4.29E+10      0.045     -29.7 ! p.w.
NHF+H=NH2+F                 2.40E+13      0.000       0.1 ! p.w.
NHF+H=NH2F                  7.49E-31     12.418   37495.2 ! p.w.
NH2+F=NHsing+HF             4.46E+09      0.561     191.6 ! p.w.
NH2+F=NH2F                  1.70E+12     -1.910    4333.3 ! p.w.
NHsing+HF=NH2F              1.13E+16     -2.400    -680.0 ! p.w.
NHsing+N2=NH+N2               4E+11       0.          0.  ! estm.
NHsing+H2=NH2+H               2E+12       0.          0.  ! estm.
NHsing+NH3=NH2+NH2            9E+13       0.          0.  ! estm.
NHsing+NF3=NF2+NHF            9E+13       0.          0.  ! (=NHsing+NH3)
NHF+H=NH+HF                 2.49E-11      7.439    8518.5 ! p.w.
NHF+NF2=HF+N2+F2            1.00E+12      0.          0.  ! Matsugi2014CNF
NHF+NF=HF+N2+F              1.45E+12      0.          0.  ! Matsugi2014CNF
NH2+F2=NH2F+F               2.90E+09      1.4      1325.  ! Matsugi2014CNF
NH+F2=NHF+F                 2.90E+09      1.4      1325.  ! Matsugi2014CNF
N+F2=NF+F                   2.90E+09      1.4      1325.  ! Matsugi2014CNF
NH2+NF2=2HF+N2              1.00E+12      0.          0.  ! Matsugi2014CNF
NH2+NF=N2+HF+H              1.45E+12      0.          0.  ! Matsugi2014CNF
NF2+NH=N2+HF+F              1.45E+12      0.          0.  ! Matsugi2014CNF
NH+NF=N2+HF                 1.50E+13      0.          0.  ! Matsugi2014CNF
NF+N=N2+F                   9.00E+11      0.5         0.  ! Matsugi2014CNF
NF2+N=N2+F+F                6.90E+13      0.          0.  ! Matsugi2014CNF
END
```

Simplified kinetic model (Chemkin format)

```
ELEMENTS
 H N F
END
SPECIES
 H H2 N NH NH2 NH3 N2 F HF NF3 NF2 NHF  NHF2 NFsing
END
THERMO ALL
   300.000  1000.000  5000.000
H                 L 6/94H   1               G   200.000  6000.00  1000.0       1
 0.25000000E+01 0.00000000E+00 0.00000000E+00 0.00000000E+00 0.00000000E+00    2
 0.25473660E+05-0.44668285E+00 0.25000000E+01 0.00000000E+00 0.00000000E+00    3
 0.00000000E+00 0.00000000E+00 0.25473660E+05-0.44668285E+00                   4
H2  REF ELEMENT   RUS 78H   2               G   200.000  6000.00  1000.0       1
 0.29328305E+01 0.82659802E-03-0.14640057E-06 0.15409851E-10-0.68879615E-15    2
-0.81305582E+03-0.10243164E+01 0.23443029E+01 0.79804248E-02-0.19477917E-04    3
 0.20156967E-07-0.73760289E-11-0.91792413E+03 0.68300218E+00                   4
N                 L 6/88N   1               G   200.000  6000.00  1000.0       1
 0.24159429E+01 0.17489065E-03-0.11902369E-06 0.30226244E-10-0.20360983E-14    2
 0.56133775E+05 0.46496095E+01 0.25000000E+01 0.00000000E+00 0.00000000E+00    3
 0.00000000E+00 0.00000000E+00 0.56104638E+05 0.41939088E+01                   4
NH                L11/89N   1H   1          G   200.000  6000.00  1000.0       1
 0.27836929E+01 0.13298429E-02-0.42478047E-06 0.78348504E-10-0.55044470E-14    2
 0.42134514E+05 0.57407798E+01 0.34929084E+01 0.31179197E-03-0.14890484E-05    3
 0.24816442E-08-0.10356967E-11 0.41894294E+05 0.18483277E+01                   4
NH2               L12/89N   1H   2          G   200.000  6000.00  1000.0       1
 0.28476611E+01 0.31428453E-02-0.89866557E-06 0.13032357E-09-0.74885356E-14    2
 0.21823916E+05 0.64718133E+01 0.42055601E+01-0.21355282E-02 0.72682021E-05    3
-0.59302799E-08 0.18067218E-11 0.21535223E+05-0.14663231E+00                   4
NH3  AMONIA       RUS 89N   1H   3          G   200.000  6000.00  1000.0       1
 2.71709692E+00 5.56856338E-03-1.76886396E-06 2.67417260E-10-1.52731419E-14    2
-6.58451989E+03 6.09289837E+00 4.30177808E+00-4.77127330E-03 2.19341619E-05    3
-2.29856489E-08 8.28992268E-12-6.74806394E+03-6.90644393E-01                   4
N2  REF ELEMENT   RUS 78N   2               G   200.000  6000.00  1000.0       1
 0.29525407E+01 0.13968838E-02-0.49262577E-06 0.78600091E-10-0.46074978E-14    2
-0.92393753E+03 0.58718221E+01 0.35309628E+01-0.12365950E-03-0.50299339E-06    3
 0.24352768E-08-0.14087954E-11-0.10469637E+04 0.29674391E+01                   4
F                 thLi97F   1               G   200.00   5000.00  1000.00      1
 2.68472005E+00-2.01550623E-04 8.73230990E-08-1.68631530E-11 1.20225312E-15    2
 8.78162871E+03 3.90029730E+00 2.41887090E+00 2.94592975E-03-8.93797264E-06    3
 9.92947105E-09-3.80146744E-12 8.75611997E+03 4.73710830E+00                   4
HF                thLi97H   1F   1          G   200.00   5000.00  1000.00      1
 3.02051188E+00 6.59427921E-04-3.46581819E-08-1.90227207E-11 2.57439346E-15    2
-3.37216641E+04 3.65476705E+00 3.48099578E+00 2.15604711E-04-6.98126549E-07    3
 8.70863939E-10-2.40504593E-13-3.39129362E+04 1.01355519E+00                   4
NF3               thLi97N   1F   3          G   200.00   5000.00  1000.00      1
 7.82521172E+00 2.73753383E-03-1.09529268E-06 2.22364403E-10-1.60848883E-14    2
-1.86685131E+04-1.49077998E+01 1.31904512E+00 2.34281290E-02-2.35120746E-05    3
 8.24365283E-09 1.94980036E-13-1.70829185E+04 1.78264736E+01                   4
NF2               thLi97N   1F   2          G   200.00   5000.00  1000.00      1
 5.67641174E+00 1.53448742E-03-6.75630227E-07 1.33193899E-10-9.63671685E-15    2
 2.16476624E+03-3.27595375E+00 3.03793228E+00 6.63330462E-03 1.60571306E-06    3
```

```
-9.87535658E-09 5.25723274E-12 2.94215307E+03 1.07299940E+01                   4
NHF              thLi97N   1H   1F   1     G   200.00   5000.00  1000.00      1
 3.71158561E+00 3.06387172E-03-1.20211924E-06 2.17526300E-10-1.46590295E-14    2
 1.21636321E+04 5.57080381E+00 4.14778046E+00-3.33523020E-03 1.76254768E-05    3
-2.05636974E-08 7.90187565E-12 1.22631218E+04 4.49092134E+00                   4
NHF2             thLi97N   1H   1F   2     G   200.00   5000.00  1000.00      1
 5.28016518E+00 4.67485094E-03-1.91039694E-06 3.55656720E-10-2.47536606E-14    2
-1.44295582E+04-1.63935123E+00 3.32084065E+00 3.50799621E-03 1.62618893E-05    3
-2.57045784E-08 1.09893746E-11-1.36320019E+04 9.90910778E+00                   4
NFsing           addhtsN   1F   1          G   200.00   5000.00  1000.00      1
 3.86263825E+00 7.43974747E-04-3.24630959E-07 7.98716895E-11-7.26947655E-15    2
 4.40083325E+04 3.16825533E+00 3.59936626E+00-2.18238902E-03 1.14120083E-05    3
-1.40082209E-08 5.53381956E-12 4.42363786E+04 5.35567652E+00                   4
END
REACTIONS
NF3+H=NF2+HF                 3.01E-03      5.188     8401.8
NF3+H=NHF2+F                 6.70E-09      6.735     8127.5
NHF2+H=NF2+H2                1.66E+05      2.628     1120.6
NF2+H=NHF+F                  7.87E+12      0.132      -69.2
NF2+H=NFsing+HF              3.25E+14     -0.616      352.7
NFsing+NH3=NHF+NH2            9.0E+13         0.        0.0
NHF+H=NH2+F                   2.4E+13         0.        0.1
NH3+F=NH2+HF                  1.5E+14         0.     1292.0
NH+H2=NH2+H                   2.1E+13         0.     15417.
NH+H=N+H2                     3.2E+13         0.       325.
NH2+N=N2+H+H                  6.9E+13         0.         0.
H+H+M=H2+M                    5.4E+18     -1.300         0.
END
```

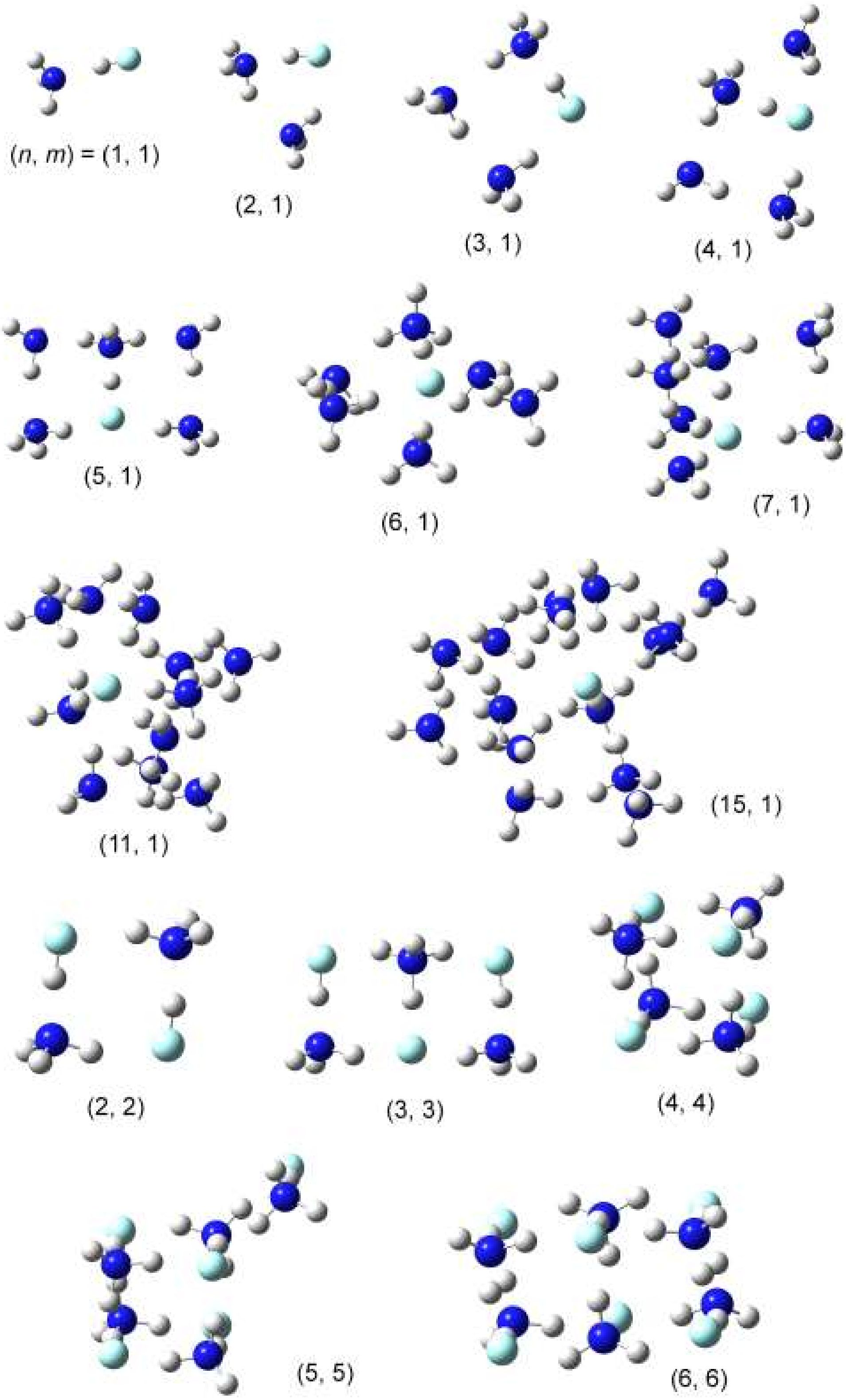

Fig. S1. Calculated structures of the $(NH_3)_n(HF)_m$ clusters.
